# Risk factors for foot ulceration in adults with end-stage renal disease on dialysis: study protocol for a prospective observational cohort study

**DOI:** 10.1186/s13047-015-0110-9

**Published:** 2015-09-18

**Authors:** Michelle R. Kaminski, Anita Raspovic, Lawrence P. McMahon, Bircan Erbas, Karl B. Landorf

**Affiliations:** Discipline of Podiatry and Lower Extremity and Gait Studies Program, School of Allied Health, La Trobe University, Melbourne, VIC 3086 Australia; Department of Podiatry, Eastern Health, Melbourne, VIC 3156 Australia; Departments of Renal Medicine and Obstetric Medicine, Eastern Health Clinical School, Monash University, Melbourne, VIC 3128 Australia; Department of Public Health, College of Science, Health and Engineering, School of Psychology and Public Health, La Trobe University, Melbourne, VIC 3086 Australia

**Keywords:** Kidney failure, Chronic, Dialysis, Foot ulcer, Risk factors, Prospective studies

## Abstract

**Background:**

Adults with end-stage renal disease treated with dialysis experience a high burden of foot ulceration and lower extremity amputation. However, the risk factors for foot ulceration in the dialysis population are incompletely understood due to the lack of high-quality prospective evidence. This article outlines the design of a prospective observational cohort study, which aims to investigate the risk factors for foot ulceration in adults on dialysis.

**Methods/Design:**

This study will recruit 430 participants with end-stage renal disease on dialysis from satellite and home-therapy dialysis units across multiple health organisations in Melbourne, Victoria, Australia. Data collection at baseline will include a participant interview, medical record review, completion of a health-status questionnaire and a non-invasive foot assessment. Twenty participants will also be recruited to a reliability study to evaluate the reproducibility of testing procedures. Primary outcome data includes: new foot ulcer(s). Secondary outcome data includes: number of new foot ulcers, time to onset of new foot ulcer(s), new lower extremity amputation(s), episodes of infection of the foot or lower extremity, episodes of osteomyelitis, foot-related hospitalisations, revascularisation procedure(s) of the lower extremity, new podiatry interventions, kidney transplantation, and mortality. Participants will be assessed at baseline, and at 12 months they will be evaluated for the primary and secondary outcomes. Multivariate Cox proportional hazards models will be used to assess predictors of new foot ulceration and time to event secondary outcomes. Logistic regression will be used for binary outcomes including prevalence of foot ulcerations.

**Discussion:**

This is the first multi-centre prospective observational cohort study to investigate risk factors for foot ulceration in adults with end-stage renal disease on dialysis. This study will improve on prior studies by using prospective methods, multi-centre recruitment, statistical methods to control for confounding variables, and a pre-specified sample size estimation. The findings can inform the design of future trials evaluating the effectiveness of clinical interventions, which may lead to improved patient outcomes in the dialysis setting.

**Electronic supplementary material:**

The online version of this article (doi:10.1186/s13047-015-0110-9) contains supplementary material, which is available to authorized users.

## Background

End-stage renal disease (ESRD) is a global public health problem characterised by a significant loss in kidney function, causing retention of metabolic waste products, salt and fluid in the body [1]. The accumulation of these waste products can become fatal unless renal replacement therapy (i.e. dialysis or kidney transplantation) is sought. It is estimated that there are more than 1.4 million people receiving renal replacement therapy worldwide [2], with the ESRD incidence rate increasing at an annual growth rate of 8 % [3].

Adults with ESRD on dialysis are at high risk for foot ulceration and subsequent lower extremity amputation [4–12]. This risk is similar to those with diabetes [5, 12, 13]. Not surprisingly, individuals who are both diabetic and dialysis-dependent have even higher rates of foot ulceration and amputation [5, 14]. Foot ulceration is a major public health concern and can have a detrimental impact on an individual’s general health, functional status and health-related quality of life [15–17]. Chronic foot ulcers progress to other serious lower extremity complications such as deep infections, and result in subsequent hospitalisation, limb loss, and foot-related mortality [5, 7, 18, 19]. In addition, the treatment and management of foot ulcers generates a considerable financial burden with increased demand on health care systems [20–22]. For example, a recent study [23] conducted in the United States reported the annual cost of diabetic foot ulceration on public and private payers is between US$9 - $13 billion, not including the costs associated with diabetes management itself.

The incidence of foot ulceration in the dialysis population is currently uncertain. However, a recent retrospective study by Lavery et al. [14] estimated a cumulative foot ulceration incidence rate of 210 per 1000 person-years in dialysis patients with coexisting diabetes. The central determinants for the development of foot ulcers in the dialysis setting are poorly understood, although meta-analyses performed in our recent systematic review found that possible risk factors include: previous foot ulceration or lower extremity amputation, peripheral arterial disease, coronary artery disease, diabetes mellitus (increasing with longer duration of disease), peripheral neuropathy, retinopathy, lower serum albumin levels and higher serum phosphorus levels [24].

Risk factors reported in the literature are frequently based on associations identified in retrospective or cross-sectional studies. To our knowledge, there are no adequately powered multi-centre prospective cohort studies that have investigated risk factors for foot ulceration in the dialysis population. Prospective studies are essential to gain greater control over data collection methods and provide a temporal sequence of events; that is, whether a variable is associated with an increase in the condition of interest [25].

With the above in mind, the aim of this article is to describe the design of a multi-centre prospective observational cohort study that will investigate risk factors for foot ulceration in adults with ESRD on dialysis.

## Methods/Design

### Ethical approval

The Human Research and Ethics Committees of La Trobe University, Eastern Health, Austin Health, and Monash Health (reference numbers: FHEC13/213, LR14/1314, LNR/14/Austin/97 and 14419X, respectively) have approved the study and all participants will provide written informed consent prior to enrolment and data collection.

### Study design

The design is a multi-centre prospective observational cohort study with a 12 month follow-up period. Initially, we performed a systematic synthesis of existing literature to quantify the major risk factors for foot ulceration and amputation in adults with ESRD treated with dialysis [24]. Our decision on which risk factors to include in this prospective observational cohort study is based not only on our systematic review findings [24], but is also informed by a comprehensive review of the diabetes literature. A 12-month follow-up period was chosen as it will provide an adequate time frame for the development of new foot ulceration [26, 27].

**Fig. 1:**
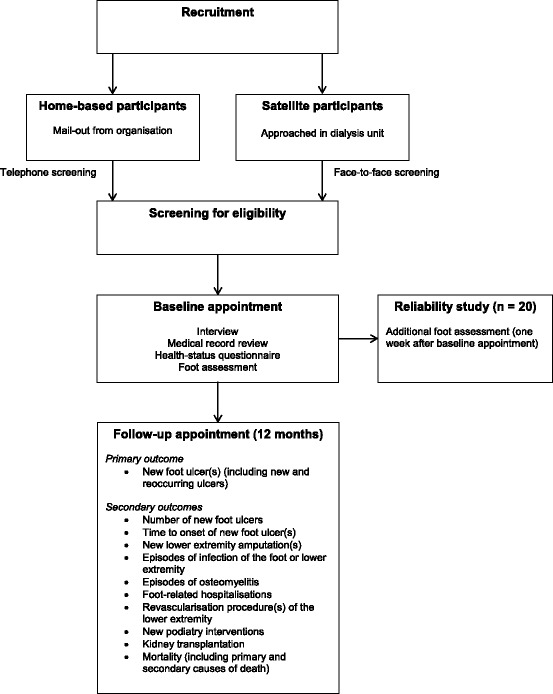
Design of study

Data collection will consist of two appointments; a baseline appointment and a follow-up appointment. One examiner (M.R.K.) will conduct the baseline and follow-up appointments on all participants. Figure 1 outlines the study design. Data to be collected at baseline will be obtained from an interview with the participant, medical record review, completion of a health-status questionnaire and a non-invasive foot assessment. A comprehensive literature review was performed to determine the most valid and reliable tools to measure suspected risk factors, for use in the foot assessment. The data collection form for the baseline data is available in Additional file 1. In addition, twenty participants will also be recruited to a reliability study to assess the repeatability of the foot assessment tools. These participants will be screened for suspected risk factors for foot ulceration on two separate occasions by the same examiner (M.R.K.), with each assessment spaced one week apart. The foot assessments to be repeated on the twenty participants in the reliability study (i.e. one week later) includes: protective sensation with the Baily Instruments Ltd® (Salford Quays, UK) Semmes-Weinstein 5.07/10 g Monofilament, vibration perception threshold with the Horwell® (Wilford, Nottingham, UK) Neurothesiometer, toe-brachial pressure index with the SysToe® (Atys Medical, Soucieu-en-Jarrest, France) system, ankle-brachial index using the Doppler ultrasound technique and first metatarsophalangeal joint range of motion with a goniometer. One examiner (M.R.K.) will perform all of the foot assessments on the participants, thus ensuring consistency and accuracy in the measurements, and reducing the chance of systematic error.

Primary and secondary outcome data will be collected at the 12 month follow-up appointment. The data collection form for the prospective data (i.e. 12-month follow-up) is available in Additional file 2. Primary outcome data includes: new foot ulcer(s) (including new and reoccurring ulcers). Secondary outcome data includes: number of new foot ulcers, time to onset of new foot ulcer(s), new lower extremity amputation(s) (including level of amputation and reason for amputation), episodes of infection of the foot or lower extremity (including type of infection), episodes of osteomyelitis, foot-related hospitalisations (including reason for admission, length of stay, and foot-related treatments/procedures received during hospital admission), revascularisation procedure(s) of the lower extremity (including type of revascularisation procedure), new podiatry interventions (e.g. nail/callus reduction, prescription of foot orthoses), kidney transplantation, and mortality (including primary and secondary causes of death).

### Participant recruitment, screening and eligibility criteria

Participants will be recruited from *satellite* dialysis units (i.e. centres where patients attend for dialysis treatment) and *home-therapy* dialysis units (i.e. dialysis treatment is performed at home) throughout Eastern Health, Austin Health and Monash Health in Melbourne, Victoria, Australia. Recruitment and collection of baseline data is anticipated to occur between January 2014 and December 2014.

Prospective participants will be identified by liaising with the renal dialysis nurses in the satellite and home-therapy dialysis units, according to the study eligibility criteria. Those attending the satellite dialysis units will be screened face-to-face, whereas respondents to the mail-out (i.e. home-therapy participants) will be screened for eligibility via telephone.

Patients attending for dialysis in the satellite units will be approached by the chief investigator (M.R.K.) during their dialysis treatment. If interested, they will be offered verbal and written information about the project and will have the opportunity to join the study at the time of meeting the researcher, or will be offered a follow-up telephone call. Alternatively, prospective participants may contact the research team at their own convenience if they are interested in participating.

Patients in the home-therapy dialysis program will be mailed a covering letter and an information package, along with an invitation to contact the research team by telephone or email if they are interested in participating. Patient lists from the home-therapy dialysis unit will be screened prior to mailing to confirm that addresses are current and to exclude any recent deaths or discharges.

To be included in the study, participants will be eligible if they:

(i) have ESRD and are clinically stable on dialysis (haemodialysis or peritoneal dialysis);
(ii) are at least 18 years of age and;
(iii) are cognitively aware (i.e. they can provide informed consent).

Participants will be excluded if they:

(i) have insufficient English skills to provide informed consent or follow instructions during the project and;
(ii) are unwilling to give informed consent to participate.

Participants’ cognition and English language proficiency (to provide informed consent or follow instructions during the project) will be confirmed by the chief investigator (M.R.K.) in collaboration with the nursing staff in the dialysis units. To determine this, prospective recruits’ understanding of what participation in the study involves will be ascertained prior to signing informed consent.

### Baseline appointment

Data will be collected at the baseline appointment via an interview with the participant and by reviewing medical records and routine blood test results. Baseline variables or factors relating to participant characteristics, comorbidities and laboratory blood test results (Table 1) were selected based on the findings of our systematic review [24] and from a comprehensive review of the diabetes literature. An average of the three latest blood test results (i.e. C-reactive protein, serum albumin, total calcium, serum phosphate, parathyroid hormone, glycated haemoglobin and haemoglobin) will be obtained at baseline for the purposes of statistical analysis.

**Table 1:** Baseline data: participant characteristics, comorbidities and laboratory blood test results

Participants characteristics	Comorbidities	Laboratory blood test results
Age (years)	Diabetes (documented diagnosis in medical history, including type and duration)	C-reactive protein (mg/L)
Sex (male or female)	Retinopathy (documented diagnosis in medical history)	Serum albumin (g/dL)
Height (m)	Known peripheral neuropathy (documented diagnosis in medical history)	Total calcium (mmol/L)
Weight (kg)	Known peripheral arterial disease or history of lower extremity revascularisation procedure (documented diagnosis in medical history or documented lower extremity revascularisation procedure e.g. angioplasty)	Serum phosphate (mmol/L)
		Parathyroid hormone (pmol/L)
		Glycated haemoglobin (%)
		Haemoglobin (g/L)
Body mass index (kg/m^2^)	Hypertension (documented diagnosis in medical history and must be requiring medication)	
Smoking history (past, current, never)	Dyslipidaemia (documented diagnosis in medical history)	
Living arrangements (living alone)	Ischaemic heart disease (documented diagnosis of ischaemic heart disease, angina, myocardial infarction or coronary bypass surgery in medical history)	
Ethnicity		
• Indigenous Australian (Aboriginal or Torres Strait Islander)		
• English	Congestive cardiac failure (documented diagnosis in medical history)	
• European		
• American		
• African	Cerebrovascular disease (documented diagnosis of cerebrovascular accident or transient ischaemic attack in medical history)	
• Asian		
• Pacific Islander		
• Other		
Cause of end-stage renal disease		
• Diabetic nephropathy	Osteoarthritis (documented diagnosis in medical history)	
• Hypertensive nephropathy		
• Chronic glomerulonephritis	Inflammatory arthritis (documented diagnosis in medical history of a type of inflammatory arthritis e.g. gout, rheumatoid arthritis, psoriatic arthropathy)	
• Polycystic kidney disease		
• Reflux nephropathy		
• Renovascular disease		
• Vasculitis		
• Unknown	Other (any other documented medical conditions)	
• Other		
Dialysis treatment		
• Haemodialysis		
• Continuous ambulatory peritoneal dialysis		
• Automated peritoneal dialysis		
Duration of dialysis (months)		

An average of the three latest blood test results (i.e. C-reactive protein, serum albumin, total calcium, serum phosphate, parathyroid hormone, glycated haemoglobin and haemoglobin) will be obtained at baseline

Previous studies have demonstrated that health-related quality of life is negatively impacted by the presence of a diabetic foot ulcer [28, 29]. Generic health status will be assessed with the short-form 36 version 2.0 health survey (SF-36v2®). The SF-36v2® has been extensively validated (construct, concurrent, content, criterion and predictive validity) and provides a reliable measure of physical and mental health in a range of populations [30–33]. The SF-36v2® is a 36 question survey that covers eight health domains that are summarised under two components, (i) a physical component summary including: physical functioning, role-physical, bodily pain and general health, and (ii) a mental component summary including: vitality, social functioning, role-emotional and mental health [34].

A non-invasive foot assessment will screen for the suspected risk factors for foot ulceration. Neurological (Table 2), arterial (Table 3), biomechanical (Table 4), footwear (Table 5) and dermatological (Table 6) assessments will be conducted. Data will also be collected on previous history of lower extremity complications (Table 7) and foot health care behaviours, including podiatry attendance (Table 8).

**Table 2:** Baseline data: neurological assessment

Neurological assessments	Equipment	Procedure	Diagnosis/study definition
Known peripheral neuropathy	N/A	Medical record review.	History of peripheral neuropathy documented in medical records.
Loss of protective sensation	Baily Instruments Ltd® (Salford Quays, UK) Semmes-Weinstein 5.07/10 g monofilament [37].	• Monofilament is first applied to participant’s hand or elbow (so that the participant knows what sensation to expect)	Failure to detect the monofilament at a specific site, even after re-testing the deficit site, in at least one foot will result in a failed test (i.e. score of <3/3 on either foot) [36].
		• Ensure the participant’s eyes are closed	
		• Monofilament applied perpendicular to the skin and held for 1–2 secs, applying sufficient force to bend or buckle the monofilament fibre [16]	
		• Monofilament applied to the plantar aspects of the hallux, first metatarsophalangeal joint and fifth metatarsophalangeal joint of both feet [36, 43]	
		• Participant is asked by the assessor to respond “yes” when they feel the monofilament	
		• This is repeated for all 3 sites on each foot (6 sites in total)	
		• Deficit sites will be re-tested once	
		• A monofilament will be not be used on more than 10 participants, without a recovery period of 24 hrs [16, 37, 91]	
Vibration perception threshold	Horwell® Neurothesiometer (Wilford, Nottingham, UK).	• Neurothesiometer is first applied to the participant’s hand or elbow (so that the participant knows what sensation to expect)	Vibration perception threshold >25 V in at least one foot will result in a failed test [40–43].
		• Participant is asked to close their eyes and to report “yes” when they first start to feel a vibratory sensation	
		• Neurothesiometer is applied to the apex of the hallux and the voltage is gradually increased until the participant perceives the vibratory sensation [40, 43]	
		• The minimum reading at which the vibratory sensation is perceived will be recorded (this is repeated 3 times and an average score is recorded for both feet) [40, 43]	
		Note: Vibration perception threshold will be measured at the base of the first, third or fifth metatarsals if there are current ulcers on the hallux, or previous amputation of the hallux [43].	

*N/A* Not applicable

**Table 3:** Baseline data: arterial assessment

Arterial assessments	Equipment	Procedure	Diagnosis/study definition
Known peripheral arterial disease and/or history of lower extremity revascularisation procedure	N/A	Medical record review.	History of peripheral arterial disease and/or lower extremity revascularisation procedure documented in medical records.
Pedal pulses	N/A	• Physical palpation of the dorsalis pedis and posterior tibial pulses on both feet with the examiners fingers (4 pulses in total) [52]	Absence of ≥2 pedal pulses will indicate peripheral arterial disease [52].
		• Pedal pulses will be recorded as ‘present’ or ‘absent’	
Toe-brachial pressure index	SysToe® (Atys Medical, Soucieu-en-Jarrest, France).	• Toe pressure measurement will be performed prior to the ankle pressure measurement to ensure arterial supply to the toes is not affected	Toe-brachial pressure index ≤0.6 will indicate peripheral arterial disease [44, 45, 53].
		• Room temperature (minimum 21–23 ± 1° C) to prevent vasoconstriction of digital arteries [92]	
		• Participants will be rested for a minimum of 15 min prior to assessment	
		• Participants to avoid use of tobacco and consumption of coffee for at least one hour prior to assessment [92, 93]	
		• Pneumatic cuff (120 x 25 mm) is placed on the proximal phalanx of hallux (i.e. proximal cuff) [47, 48]	
		Note: If hallux is absent, a 90 x 15 mm digital cuff will be used on the second toe [48, 49].	
		• Double-sided tape is applied to sensor [47]	
		• Sensor is positioned on the plantar aspect of the hallux (or second toe) and secured with another pneumatic cuff (i.e. distal cuff) [47, 48]	
		• Turning the SysToe® device on will cause an automated sequence involving inflation of the distal cuff, then inflation of the proximal cuff, followed by rapid deflation of the distal cuff and slower deflation of the proximal cuff (3 mm Hg s^−1^) [47, 48]	
		• The return of blood perfusion (measured by the proximal cuff) will be recorded as the toe systolic pressure [47, 48]	
		• Toe pressure assessment is repeated for contralateral side (if appropriate)	
		• Toe-brachial pressure index value is calculated by dividing the toe systolic pressure by the highest (or available) brachial systolic pressure	
		• Toe brachial pressure index value calculated separately for left and right lower limbs	
		Note: Brachial systolic pressures obtained in the ankle-brachial pressure index assessment will be used to calculate the toe-brachial pressure index value.	
Ankle-brachial pressure index	Hadeco Bidop ES100V3 Bi-Directional Doppler Complete with LCD Display and 8 MHz Probe.	• Room temperature (minimum 21–23 ± 1° C) to prevent vasoconstriction of digital arteries [92]	Ankle-brachial pressure index ≤0.9 will indicate peripheral arterial disease [44, 45, 53, 54].
	Erka® ‘Switch’ Sphygmomanometer and cuff.	• Participants will be rested for a minimum of 15 min prior to assessment	Ankle-brachial pressure index >1.3 or non-compressible arteries (i.e. >240 mm Hg) will indicate arterial calcification [44, 54].
		• Participants to avoid use of tobacco and consumption of coffee for at least one hour prior to assessment [92, 93]	
		• Brachial cuff is applied 3 cm above the cubital fossa	
		• Brachial pulse located via palpation	
		• Doppler ultrasound conducting gel is applied to the skin [92]	
		• Doppler probe is applied at a 45° angle to the skin [92], in the direction of the arterial blood flow	
		• Cuff is inflated to 20–30 mm Hg beyond the last audible signal and then slowly deflated until the audible signal returns [92]	
		• Repeated for contralateral side (if appropriate)	
		• Brachial systolic pressure(s) recorded	
		Note: In the case of an arteriovenous fistula (i.e. vascular access for haemodialysis treatment) the brachial systolic pressure will be measured from the arm free of the fistula [45].	
		• Ankle cuff is applied 3 cm above the medial malleolus	
		• Dorsalis pedis and posterior tibial pulses are located via palpation	
		• Doppler ultrasound conducting gel is applied to the skin [92]	
		• Doppler probe is applied at a 45° angle to the skin [92], in the direction of the arterial blood flow	
		• Cuff is inflated to 20–30 mm Hg beyond the last audible signal and then slowly deflated until the audible signal returns [92] (maximum 240 mm Hg). Process is repeated for both the dorsalis pedis and posterior tibial pulses	
		• Repeated for contralateral side (if appropriate).	
		• The highest of the two systolic pressure values obtained from the dorsalis pedis and posterior tibial pulses will be recorded [92]	
		Note: If the pressure needs to be repeated, the examiner will wait one minute before re-inflating the cuff [92].	
		• Ankle-brachial pressure index value is calculated by dividing the highest ankle systolic pressure (i.e. highest value between dorsalis pedis and posterior tibial pulses) by the highest (or available) brachial systolic pressure	
		Note: Ankle-brachial pressure index value is calculated separately for left and right lower limbs.	

*N/A* Not applicable

**Table 4:** Baseline data: biomechanical assessment

Biomechanical assessments	Equipment	Procedure	Diagnosis/study definition
Foot deformity	The Manchester Scale [57].	• The presence of hammer/claw toes, hallux abducto valgus, bony prominences (e.g. prominent metatarsal heads), Charcot neuroarthropathy and any other foot deformities (e.g. forefoot pad atrophy) will be assessed visually [57]	Foot deformity will be recorded with the presence of ≥1 foot deformity on either foot.
		• Hallux abducto valgus will be graded in accordance with the Manchester Scale (no deformity = 1, mild deformity = 2, moderate deformity = 3, severe deformity = 4) [57, 58]	
		• Foot deformity will be recorded as ‘present’ or ‘absent’	
Range of motion (1^st^ metatarsophalangeal joint)	Goniometer.	• Passive range of dorsiflexion at the 1^st^ metatarsophalangeal joint will be measured using goniometry with the ‘static non-weightbearing technique 1’ described by Hopson et al. [60]	Range of motion <65° indicates limited joint mobility of the first metatarsophalangeal joint [60].
Plantar pressures	Tekscan Matscan® system (Tekscan Inc, South Boston, MA, USA).	• Plantar pressures will be assessed during level barefoot walking with the Tekscan Matscan® system [64, 94]	Mean peak plantar pressures will be investigated to determine whether they are predictive of foot ulceration.
	5.7 mm thick floor mat (436 × 369 mm), 2288 resistive sensors (1.4 sensors/cm^2^), dynamic events captured with scan rates of 440 Hz.	• The two-step gait initiation protocol will be used, with the technique as described by Zammit et al. [64], except that both feet will be assessed	
	FootMat™ 7.0 software (Tekscan Inc, South Boston, MA, USA).	• The mat will be calibrated for each patient using that patient’s own weight before each testing session	
		• Peak plantar pressure will be measured at seven regions of the foot, including the heel, midfoot, first metatarsophalangeal joint, second metatarsophalangeal joint, 3–5 metatarsophalangeal joints, hallux and lesser toes [64]	
		• The mean peak plantar pressure values of the three trials of each foot will be used for final data analysis [64, 95]

**Table 5:** Baseline data: footwear assessment

Footwear assessments	Procedure	Diagnosis/study definition
Fit, general features, style and condition	• The fit (length, width, depth), general features (fixation, forefoot sole flexion point, heel height, materials), style and condition of footwear will be assessed based on the footwear assessment tool described by Barton et al. [65]	Footwear will be deemed inappropriate if there are any issues with shoe fit, inappropriate style or condition.

**Table 6:** Baseline data: dermatological assessment

Dermatological assessments	Procedure	Diagnosis/study definition
Skin pathology	• The presence of hyperkeratosis (callus), heloma dura (corns), uraemic pruritus, xerosis, calciphylaxis and other skin pathologies will be assessed visually [18, 66, 67, 70–72]	Skin pathology will be recorded with the presence of ≥1 skin pathology on either foot.
	• Severity of xerosis will be graded in accordance with the Xerosis Severity Scale (mild = 1–2, moderate = 3–4, severe = 5–6) [66, 67]	
	• Skin pathology will be recorded as ‘present’ or ‘absent’	
Nail pathology	• The presence of half-and-half nail, absent lunula, onychomycosis, onychocryptosis (ingrown nail), onychauxis (thickened nail) and other nail pathologies will be assessed visually [70, 71]	Nail pathology will be recorded with the presence of ≥1 nail pathology on either foot.
	• Nail pathology will be recorded as ‘present’ or ‘absent’

**Table 7:** Baseline data: history of lower extremity complications

Lower extremity complication	Procedure	Diagnosis/study definition
Foot ulceration	• Past or current foot ulcers will be determined by self-report, observation and medical record review	A foot ulcer will be defined as a ‘full thickness skin break that is distal to the ankle joint, and may extend into or through the dermis and involve deeper structures such as bones, tendons, joint capsules and ligaments’ [41, 55, 73].
	• The location, type and duration of a current foot ulcer will be recorded	
Lower extremity amputation	• Past lower extremity amputations will be determined by self-report, observation and medical record review	A lower extremity amputation will be defined as a ‘complete loss of any part of the lower extremity [74], including any digit, partial foot amputation or higher’.
	• Lower extremity amputations will be classified as minor (below ankle) or major (above ankle)	Lower extremity amputations resulting from trauma or the presence of a tumour will not be recorded.
		A major amputation will be classified as a loss of limb above the ankle, or minor amputation if below the ankle [74, 75].

**Table 8:** Baseline data: foot health care behaviours and podiatry attendance

Foot health care behaviours and podiatry attendance	Procedure	Diagnosis/study definition
Foot health care behaviours	• Foot health care behaviours will be investigated via participant interview	Foot health care behaviours will be considered ‘poor’ if the participant answers “no” to ≥3 questions.
	• Participants will be asked to respond “yes” or “no” to the following questions:	
	(i) Do you inspect your feet daily?	
	(ii) Do you avoid walking barefoot?	
	(iii) Are you able to reach your feet?	
	(iv) Do you treat your own nails and skin lesions?	
	(v) Have you ever seen a podiatrist before?	
Podiatry attendance	• Podiatry attendance will be investigated via participant interview	Podiatry attendance will be recorded as the number of visits per year.
	• Participants will be asked: How many times have you seen a podiatrist in the last 12 months?	

#### Neurological assessment

The presence of peripheral neuropathy will be assessed using a Baily Instruments Ltd® (Salford Quays, UK) Semmes-Weinstein 5.07/10 g Monofilament and a Horwell® (Wilford, Nottingham, UK) Neurothesiometer, based on the recommendations of the American Diabetes Association [35]. These tests will evaluate loss of protective sensation and vibration perception threshold, respectively.

A recent systematic review reported that a failed Semmes-Weinstein monofilament test is a significant and independent predictor for subsequent foot ulceration in patients with diabetes [36]. A systematic review of validation studies found that testing of loss of protective sensation with a 10 g monofilament has reported sensitivity of 57 to 93 % and specificity of 75 to 100 % for detecting diabetic neuropathy [36]. The Baily Instruments Ltd® monofilament was selected for use in this study as it is one of the most accurate monofilaments to produce a 10 g buckling force (100 % buckling within ±1.0 g of 10 g) [37]. The plantar aspects of the hallux, first and fifth metatarsals will be assessed [38, 39].

The vibration perception threshold will be evaluated as it can identify those with less severe forms of neuropathy (i.e. before loss of protective sensation is evident clinically) [40]. A review of validation studies found that testing of vibration perception threshold with either a Neurothesiometer or Biothesiometer has reported sensitivity (77 to 100 %) and specificity (73 to 81 %) ranges for detecting diabetic neuropathy [40]. A vibration perception threshold of >25 V (tested on the apex of the hallux) in at least one foot will be used as the cut off value in this study, as it has been associated with a high cumulative risk of neuropathic ulceration [41, 42].

Peripheral neuropathy will be recorded if any of the following are present:

- Documentation of known peripheral neuropathy in the medical records;
- Monofilament score of <3/3 (either foot) [36];
- Vibration perception threshold >25 V (either foot) [40–43].

#### Arterial assessment

The presence of peripheral arterial disease will be assessed by palpating pedal pulses (i.e. dorsalis pedis and posterior tibial) and by calculating the toe-brachial pressure index and the ankle-brachial pressure index bilaterally. Symptoms for peripheral arterial disease (i.e. intermittent claudication and rest pain) will not be assessed in this study, as it is likely that there is a high prevalence of *asymptomatic* peripheral arterial disease in this population, due to a high co-prevalence of diabetes, infections and neuropathy [44]. A combination of the toe-brachial pressure index and ankle-brachial pressure index measurements will allow for a more accurate representation of the prevalence of peripheral arterial disease, due to the known high prevalence rates of medial arterial calcification in dialysis patients [45, 46].

As per the recommendations of Leskinen et al. [45], toe-brachial pressure indices ≤0.6 and/or ankle-brachial pressure indices ≤0.9 will indicate the presence of peripheral arterial disease. The SysToe® (Atys Medical, Soucieu-en-Jarrest, France) system was chosen to determine the toe systolic pressure as it has been shown to be a valid and reliable clinical assessment tool (Intraclass Correlation Coefficients, ICC = 0.89 and 0.91 for the right and left sides respectively) [47–49]. The Doppler ultrasound technique will be used to assess the ankle and brachial systolic pressures, as it is known to be a valid and reliable assessment method in the detection of peripheral arterial disease [50, 51]. In addition, ankle-brachial pressure indices ≤0.9 have been reported to have a high sensitivity (75 %) and specificity (86 %) for the diagnosis of peripheral arterial disease in pooled meta-analyses [50].

Peripheral arterial disease will be recorded if any of the following are present:

- Documentation of known peripheral arterial disease in the medical records and/or history of lower extremity revascularisation procedure;
- Absence of ≥2 pedal pulses after palpating the dorsalis pedis and posterior tibial pulses on both feet [52];
- Toe-brachial pressure index ≤0.6 (either foot) [44, 45, 53] and/or ankle-brachial pressure index ≤0.9 (either foot/lower extremity) [44, 45, 50, 53, 54].

Arterial calcification will be recorded if any of the following are present:

- Ankle-brachial pressure index >1.3 (either foot/lower extremity) [44, 54];
- Non-compressible peripheral arteries (i.e. systolic pressure >240 mm Hg).

#### Biomechanical assessment

Anomalies of foot structure have been found to be predictive of foot ulceration in prospective studies [41, 55, 56]. The grading of hallux valgus will be evaluated using the Manchester Scale [57], as it provides a valid representation of the degree of hallux valgus deformity [58]. Intra- and inter-rater reliability have been found to be excellent, with kappa (κ) values of 0.77 and 0.86, respectively [57]. Foot deformity will be recorded if any of the following are present:

- Hallux abducto valgus (graded in accordance with the Manchester Scale) [35, 57];
- Hammer/claw toes [35];
- Bony prominences (e.g. prominent metatarsal heads);
- Charcot neuroarthropathy [35];
- Other (e.g. forefoot pad atrophy).

Limited joint range of motion (e.g. first metatarsophalangeal joint) has also been associated with an increased risk of foot ulceration in prospective and case–control studies [55, 56, 59].

Dorsiflexion range of motion of the first metatarsophalangeal joint will be assessed in accordance with the ‘static non-weightbearing technique 1’ described by Hopson et al. [60]. This technique has been shown to be a reliable clinical measurement (ICC = 0.95). Limited range of motion at the first metatarsophalangeal joint will be documented if:

- Passive, non-weightbearing dorsiflexion is <65° (either foot) [60].

Elevated peak plantar pressures have been found to be a statistically significant predictor of diabetic foot ulceration in prospective studies [61–63]. Peak plantar pressures will be assessed during level barefoot walking using a two-step gait initiation protocol [64] with the Tekscan Matscan® system (Tekscan Inc, South Boston, MA, USA). Peak plantar pressures will be measured at seven regions of the foot, including the heel, midfoot, first metatarsophalangeal joint, second metatarsophalangeal joint, third to fifth metatarsophalangeal joints, hallux, and lesser toes [64]. The mean peak plantar pressure values of the three trials (of each foot) will be used for the purposes of analysis.

#### Footwear assessment

Minor trauma that results in tissue damage or a break in the cutaneous barrier of the skin is most commonly due to ill-fitting or inappropriate footwear, which is frequently implicated in the development of foot ulcers due to rubbing and repetitive trauma [41, 55]. Reiber et al. [55] found that a minor traumatic event was one of the main component causes for the development of foot ulcers, which was present in 77 % of the foot ulcer pathways. In addition, pressure from footwear caused the minor traumatic event in 55 % of the participants [55]. Assessment of footwear fit, general features, type and condition is based on the recommendations of the validated footwear assessment tool described by Barton et al. [65]. Footwear will be deemed inappropriate if any of the following are present:

- Poor shoe fit (i.e. length, width and depth);
- Inappropriate shoe style (i.e. for foot type, activity, foot problems etc.) or;
- Poor shoe condition [65].

#### Dermatological assessment

Skin and nail pathologies have been found to be predictive of foot ulceration in people with diabetes in prospective studies [27, 55]. The grading of xerosis severity will be evaluated using the Xerosis Severity Scale, which has been used in several randomised trials [66–68]. Skin pathology will be recorded if any of the following are present:

- Hyperkeratosis [16, 69];
- Heloma dura [16, 69];
- Uraemic pruritus [70, 71];
- Xerosis (graded in accordance with the Xerosis Severity Scale) [67];
- Calciphylaxis [18, 72];
- Other.

Nail pathology will be recorded if any of the following are present:

- Half-and-half nail [70, 71];
- Absent lunula [70, 71];
- Onychomycosis [27, 69];
- Onychocryptosis [69];
- Onychauxis [69];
- Other.

#### Foot health care behaviours and podiatry attendance

Foot health care behaviours will be considered ‘poor’ if the participant answers “no” to three or more of the following questions:

- Do you inspect your feet daily?
- Do you avoid walking barefoot?
- Are you able to reach your feet?
- Do you treat your own nails and skin lesions? (e.g. calluses or corns)
- Have you ever seen a podiatrist before?

Regular podiatry attendance will be determined with the following question:

- How many times have you seen a podiatrist in the last 12 months?

### Follow-up appointment

The follow-up appointment will occur 12 months after the baseline appointment. Satellite participants will be followed-up face-to-face in the dialysis units, whereas home-therapy participants will be contacted via telephone. Prior to the follow-up appointment, participant medical records will be screened for any recent deaths or discharges. This is to ensure that the families of participants who died during the study are not contacted.

### Primary outcome

The primary outcome in this prospective observational cohort study will be:

- new foot ulcer(s) (including new and reoccurring ulcers).

A foot ulcer will be defined as a ‘full thickness skin break that is distal to the ankle joint, and may extend into or through the dermis and involve deeper structures such as bones, tendons, joint capsules and ligaments’ [27, 73]. Current foot ulcers (i.e. ulcers present at baseline appointment) that healed and re-ulcerated during the follow-up period will also be recorded as new foot ulcers. At the 12-month follow-up appointment, participants will self-report whether they developed a new foot ulcer since their baseline appointment. This information will be verified by reviewing participants’ medical records. All new foot ulcers documented at the follow-up appointment will be differentiated into ‘new’ and ‘reoccurring’ ulcers. All medical histories of participants will be reviewed to determine whether any new foot ulcers occurred during the 12-month follow-up period.

### Secondary outcomes

Secondary outcome data will also be collected at the 12 month follow-up appointment including:

- number of new foot ulcers;
- time to onset of new foot ulcer(s);
- new lower extremity amputation(s) (including level of amputation and reason for amputation);
- episodes of infection of the foot or lower extremity (including type of infection);
- episodes of osteomyelitis;
- foot-related hospitalisations (including reason for admission, length of stay and foot-related treatments/procedures received during hospital admission);
- revascularisation procedure(s) of the lower extremity (including type of procedure);
- new podiatry interventions (e.g. nail reduction, prescription of foot orthoses);
- kidney transplantation and;
- mortality (including primary and secondary causes of death).

All secondary outcomes will be self-reported by participants and then verified by reviewing medical records. All medical histories of participants will be reviewed to determine whether any of the secondary outcomes occurred during the 12-month follow-up period. The time to onset of new foot ulcer(s) will be defined as the ‘number of days between the baseline appointment and the development of a new foot ulcer’. Medical records will be referred to for the first documented encounter of the new foot ulcer and this date will be recorded. A lower extremity amputation will be defined as a ‘complete loss of any part of the lower extremity [74], including any digit, partial foot amputation or higher’. Lower extremity amputations resulting from accidental trauma unrelated to ESRD or the presence of a tumour will not be recorded. Lower extremity amputations will be classified as major or minor. A major amputation will be documented if there is loss of limb above the ankle, or minor amputation if below the ankle [74, 75]. Mortality data will also be collected at the follow-up appointment. Date of death (including primary and secondary causes of death) will also be recorded from reviewing patient medical histories. For those participants that die during the study period, hospital medical records will be reviewed to assess for the primary and secondary outcomes up until the date of death.

### Sample size

Prospective sample size calculations were performed using Stata 11 Data Analysis and Statistical Software (StataCorp LP, Texas, USA) for the following predictor variables; diabetes mellitus and peripheral neuropathy. Based on these calculations, a sample of 430 participants will provide 80 % power to detect a clinically worthwhile difference of 15 % in the incidence of foot ulceration between those with and without diabetes. This sample size will also provide 80 % power to detect a clinically worthwhile difference of 10 % in the incidence of foot ulceration between those with and without peripheral neuropathy. This sample size also allows for a 20 % loss to follow-up.

### Data handling and statistical analysis

Data will be entered into a Microsoft Excel® spread sheet for the development of an analytical file. A predefined statistical analysis plan will be established between an experienced biostatistician (B.E.) and the research team prior to commencing the analysis. Baseline sociodemographic, health-related quality of life and clinical characteristics of participants will be calculated and expressed as mean ± standard deviation or median (25^th^ to 75^th^ percentile) for continuous variables and number (proportion) for categorical variables. For comparison of baseline variables between different groups (for example, foot ulcer or no foot ulcer), independent samples *t*-tests will be calculated for continuous variables and chi-square (*χ*^2^) tests for categorical variables. Unadjusted foot ulcer incidence rates will be calculated for number of events per 1000 person-years. Multivariate Cox proportional hazards models will be used to assess predictors of new foot ulceration and time to event secondary outcomes. Logistic regression will be used for binary outcomes, such as prevalence of foot ulcerations.

Base regression models will be developed to fit each risk factor one at a time with other variables including baseline demographics and possible confounders as described in Table 9 [9, 76, 77], and statistical significance will be assessed. As a sensitivity analysis, we will also use forward selection when building models with *P*-values set at 0.1. Confounders will be retained if they change the estimated associations between risk factors and the outcome by 10 % or more, or are significant at the 5 % level in adjusted models [78]. After a number of iterations, all risk factors will then be combined in a multivariable regression model. Possible interactions will also be assessed. Strata specific analysis of regression models using diabetes (yes/no) and sex (male/female) will be conducted to assess variables for possible effect modification. Interaction terms will then be included in the regression models if there is evidence of an effect modification from any of these variables. Risk estimates will be presented as hazard ratios and/or odds ratios (depending on the regression model) with corresponding 95 % confidence intervals (CIs). Statistical significance will be set at the conventional level of *P* < 0.05.

**Table 9:** Risk factors and potential confounding variables

Continuous variables	Categorical variables
Participant characteristics	Participant characteristics
• **Age**	• **Male sex**
• **Body mass index**	• **Current smoker**
Health-related quality of life (SF-36v2®)	• **Living alone**
• Physical Component Score	Comorbidities
• Mental Component Score	• **Diabetes mellitus**
Comorbidities	• **Retinopathy**
• **Duration of diabetes**	• **Peripheral neuropathy**
Dialysis-related variables	• **Peripheral arterial disease**
• **Duration of dialysis**	• **Arterial calcification**
Laboratory blood tests	• **Hypertension**
• C-reactive protein	• **Dyslipidaemia**
• Serum albumin	• **Ischaemic heart disease**
• Total calcium	• **Congestive cardiac failure**
• Serum phosphate	• **Cerebrovascular disease**
• Parathyroid hormone	• Osteoarthritis
• Glycated haemoglobin (HbA1c)	• Inflammatory arthritis
• Haemoglobin	Lower extremity complications
Other	• **Previous foot ulceration**
• Peak plantar pressures	• **Current foot ulcer present at baseline**
	• **Previous lower extremity amputation**
	Other
	• Reduced range of motion of the 1^st^ metatarsophalangeal joint
	• **Foot deformity**
	• **Inappropriate/ill-fitting footwear**
	• Skin pathology
	• Nail pathology
	• Poor foot health care behaviours
	• Regular podiatry attendance

Potential confounding variables that will be considered in the regression models are boldface

Intra-examiner reliability of the assessor (M.R.K.) will be evaluated using ICCs for continuous data and the weighted kappa (κ) statistic for ordinal data. To assess the strength of linear correlation between the two measurements (i.e. to detect random and systematic errors) [25], ICCs and corresponding 95 % CIs of the type (3, 1) will be calculated for single measures and type (3, *k*) for average measures. ICC values >0.75 indicate good reliability, ICCs ranging from 0.50 to 0.75 imply moderate reliability and ICCs <0.50 suggest poor reliability [25]. The 95 % Limits of Agreement will also be calculated to examine the level of agreement between the two measurements [25, 79]. Absolute agreement in addition to linear weighted kappa will be calculated for ordinal data to determine the proportion of the total amount of agreement between the two measurements that is not explained by chance [80, 81]. Weighted kappa values >0.8 represent excellent agreement, >0.6 substantial levels of agreement, 0.4 to 0.6 moderate agreement and <0.4 poor to fair agreement [25].

Data analysis will be undertaken using IBM SPSS version 22.0 or later (IBM Corp, Somers, NY, USA), Stata 11 Data Analysis and Statistical Software (StataCorp LP, Texas, USA), QualityMetric Health Outcomes™ Scoring Software 4.5.1 and FootMat™ 7.0 Software (Tekscan Inc, South Boston, MA, USA).

## Discussion

Foot ulceration is a major public health problem that can have a negative impact on an individual’s general health, functional status and health-related quality of life [15–17]. Despite the high prevalence of foot ulceration in the dialysis population, there is a paucity of high-quality prospective studies that have investigated the risk factors associated with this condition. Existing risk factor studies [5, 6, 8–10, 12, 82–88] have been limited by small sample sizes, inadequate control for confounding variables, do not encompass a full range of risk factors, and the majority are either cross-sectional or retrospective. Moreover, these study designs do not examine for a temporal sequence of events, so it is difficult to conclude whether these risk factors are associated with an increase in foot ulceration. Importantly, our study protocol has been developed to improve on the design of previous studies.

Our prospective observational cohort study has been designed to investigate the risk factors for foot ulceration in adults with ESRD on dialysis. We have chosen to evaluate the risk factors for foot ulceration specifically in dialysis patients, as these individuals have been found to be at high risk for foot complications [7, 9, 89, 90]. People with or without diabetes will be recruited into the study. This cohort study will improve on prior studies by using prospective methods, multi-centre recruitment, statistical methods to control for confounding variables, and a pre-specified sample size estimation.

The study will investigate participant characteristics, medical history, blood tests, health-related quality of life and foot assessment data to assess whether any predict important clinical outcomes including: new foot ulcer(s), (primary outcome), number of new foot ulcers, time to onset of new foot ulcer(s), new lower extremity amputation(s), episodes of infection of the foot or lower extremity, episodes of osteomyelitis, foot-related hospitalisations, revascularisation procedure(s) of the lower extremity, and mortality (secondary outcomes).

There are several strengths of our study. Firstly, the baseline data collection form (Additional file 1) is based on the findings of our systematic review [24] and from a comprehensive review of the diabetes literature. Secondly, one examiner (M.R.K.) will perform all of the foot assessments on the participants, thus ensuring consistency and accuracy in the measurements, and reducing the chance of systematic error. Thirdly, we will assess the reliability of the foot assessment tools. Twenty participants will be included in a reliability study, which will evaluate the reproducibility of the testing procedures. A one week test-retest interval was chosen as it is sufficient time to avoid fatigue, learning or memory effects, and will also avoid genuine changes in the measurement variables [25]. Fourthly, the study protocol has been pragmatically designed to encompass a full range of risk factors and to ensure that the findings can be generalised and applied to clinical practice (if there are significant risk factors for foot ulceration identified). Finally, a prospective sample size calculation was performed to ensure that the study is adequately powered.

There are also a few potential limitations to our study. While the examiner will avoid providing feedback and education to participants during their foot assessment, it is not possible to control for all other potential confounding treatments or interventions that participants may receive from other sources during the study period. For example, a participant may receive an intervention that directly affects their risk of developing a foot ulcer or not (e.g. prescription of pressure offloading foot orthoses). In addition, participants involved in this study may become more aware of their own foot health as a result of their participation, and may therefore change their behaviour during the study period (e.g. start to perform daily foot checks, seek podiatry treatment, etc.). Ethical considerations may also affect the final outcomes of this study. For example, the researcher will report any abnormal findings from the individual foot assessments (e.g. new foot ulcer or presence of peripheral arterial disease) to the medical teams in the dialysis units. As such, participants may receive new interventions as a result (e.g. lower extremity revascularisation procedure), which may have a direct effect on the final outcomes. Lastly, as this cohort is made up of severely ill participants, mortality and loss to follow-up due to comorbidities could be potentially higher than the expected 20 %. If this is exceeded, we will address this issue by using imputation techniques for missing data in regression models.

## Conclusion

This is the first multi-centre prospective observational cohort study to investigate risk factors for foot ulceration in adults with ESRD treated with dialysis. In addition, we have pre-specified our sample size to ensure clinically and statistically meaningful results. The study will improve on prior studies by using prospective methods, multi-centre recruitment, statistical methods to control for confounding variables, and a pre-specified sample size estimation. The results will provide high-level evidence, and a temporal sequence of events for risk factors contributing to the development of foot ulceration in the dialysis population. The identification of potentially modifiable risk factors can inform the design of future trials investigating the effectiveness of clinical interventions to reduce the burden of lower limb disease in adults on dialysis.

## Additional files

Additional file 1:
**Data collection form: Baseline data.** Data collection form is designed to screen for potential risk factors for foot ulceration at baseline. (PDF 159 kb) Additional file 2:
**Data collection form: Prospective data (12-month follow-up).** Data collection form is designed to obtain primary and secondary outcome data at the 12-month follow-up. (PDF 145 kb)

## Abbreviations

ESRD: End-stage renal disease
SF-36v2®: Short-form 36 version 2.0 health survey
ICC: Intraclass correlation coefficient
CI: Confidence interval
N/A: Not applicable
