# Supplementary material for: Risk factors for foot ulceration in adults with end-stage renal disease on dialysis: study protocol for a prospective observational cohort study
Source: J Foot Ankle Res. 2015 Sep 18;8:53. doi: 10.1186/s13047-015-0110-9 (PMC4575467; doi:10.1186/s13047-015-0110-9)
Supplement: Additional file 1: — Data collection form: Baseline data. Data collection form is designed to screen for potential risk factors for foot ulceration at baseline. (PDF 159 kb) [file 13047_2015_110_MOESM1_ESM.pdf]

Data Collection Form: Baseline Data

1. Participant Characteristics

Date of birth: / / **Sex:** Male ☐1 Female ☐2

Height: ________m **Weight:** ________kg **Body mass index:** ________ (kg/m²)

Smoking history: Past ☐1 Current ☐2 Never ☐3

Living alone: Yes ☐1 No ☐2

Ethnicity:
- ☐1 Indigenous Australian (Aboriginal or Torres Strait Islander)
- ☐2 English
- ☐3 European
- ☐4 American
- ☐5 African
- ☐6 Asian
- ☐7 Pacific Islander
- ☐8 Other____________

Cause of ESRD:
- Diabetic nephropathy ☐1
- Hypertensive nephropathy ☐2
- Chronic glomerulonephritis ☐3
- Polycystic kidney disease ☐4
- Reflux nephropathy ☐5
- Renovascular disease ☐6
- Vasculitis ☐7
- Unknown ☐8
- Other____________ ☐9

Dialysis treatment: Haemodialysis ☐1 CAPD ☐2 APD ☐3

Dialysis start date: ____________ **Duration of dialysis (months):** ____________

2. Comorbidities

Diabetes: Yes ☐1 No ☐2

Type of diabetes: Type 1 ☐1 Type 2 ☐2

Date of diagnosis: ____________ **Duration of diabetes (months):** ____________

**Retinopathy:**	Yes	☐1	No	☐2
**Known peripheral neuropathy:**	Yes	☐1	No	☐2
**Known peripheral arterial disease:**	Yes	☐1	No	☐2
**Lower extremity revascularisation procedure:**	Yes	☐1	No	☐2
**Hypertension (requiring medication):**	Yes	☐1	No	☐2
**Dyslipidaemia:**	Yes	☐1	No	☐2
**Ischaemic heart disease:**	Yes	☐1	No	☐2
**Congestive cardiac failure:**	Yes	☐1	No	☐2
**Cerebrovascular disease:**	Yes	☐1	No	☐2
**Osteoarthritis:**	Yes	☐1	No	☐2
**Inflammatory arthritis:**	Yes	☐1	No	☐2

Other ____________

3. Laboratory Results

**Parameter**	**Blood test 1**	**Blood test 2**	**Blood test 3**	**Average result**
C-Reactive Protein (mg/L)				
Serum Albumin (g/dL)				
Total Calcium (mmol/L)				
Serum Phosphate (mmol/L)				
Parathyroid Hormone (pmol/L)				
Glycated haemoglobin (%)				
Haemoglobin (g/L)				

4. Risk Factors

4.1 History of Lower Extremity Complications

4.1.1 Past Foot Ulceration

**Left foot**	Yes	☐ 1	No	☐ 2
**Right foot**	Yes	☐ 1	No	☐ 2

4.1.2 Current Foot Ulceration

**Left foot**	Yes	☐ 1	No	☐ 2
**Right foot**	Yes	☐ 1	No	☐ 2

4.1.2.1 Location

Left Foot

- ☐ 1 Dorsal toes
- ☐ 2 Plantar toes, forefoot, and midfoot
- ☐ 3 Dorsal foot
- ☐ 4 Heel
- ☐ 5 Multiple areas of the foot

Right Foot

- ☐ 1 Dorsal toes
- ☐ 2 Plantar toes, forefoot, and midfoot
- ☐ 3 Dorsal foot
- ☐ 4 Heel
- ☐ 5 Multiple areas of the foot

4.1.2.2 Type

Left Foot

(Based on results of neurovascular assessment)

- Neuropathic ☐ 1
- Neuro-ischaemic ☐ 2
- Ischaemic ☐ 3
- Other ☐ 4

Right Foot

(Based on results of neurovascular assessment)

- Neuropathic ☐ 1
- Neuro-ischaemic ☐ 2
- Ischaemic ☐ 3
- Other ☐ 4

4.1.2.3 Duration

Left foot
Duration (months): ____________

Right foot
Duration (months): ____________

4.1.3 Lower Extremity Amputation (LEA)

Left lower extremity

Minor (below ankle) ☐1 Major (above ankle) ☐2 N/A ☐3

Location of LEA: ____________

Right lower extremity

Minor (below ankle) ☐1 Major (above ankle) ☐2 N/A ☐3

Location of LEA: ____________

4.2 Neurological Assessment

4.2.1 Vibration Perception Threshold (Neurothesiometer)

	Trial 1 Volts (v)	Trial 2 Volts (v)	Trial 3 Volts (v)	Average Volts (v)
Left Foot				
Right Foot				

Note. Vibration perception threshold >25 V indicates peripheral neuropathy.

4.2.2 Protective Sensation (Semmes-Weinstein 5.07/10g Monofilament)

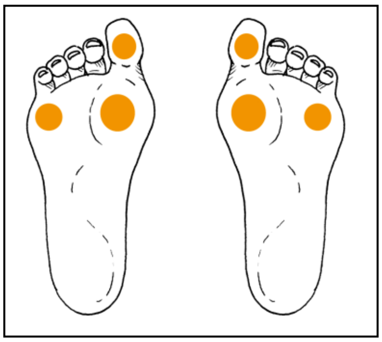

	Sites (Plantar hallux, plantar 1st MTPJ and plantar 5th MTPJ)
Left Foot	/3
Right Foot	/3

Note. Inability to detect the monofilament at ≥1 site(s) indicates peripheral neuropathy.

4.3 Arterial Assessment

4.3.1 Pedal Pulses

	Dorsalis Pedis	Posterior Tibial
**Left Foot**		
**Right Foot**		

Note. P= palpable, NP= not palpable. Absence of ≥2 pedal pulses on both feet indicates peripheral arterial disease.

4.3.2 Ankle-Brachial Pressure Index (ABPI) and 4.3.3 Toe-Brachial Pressure Index (TBPI)

	Left	Right
**Toe Systolic Pressure (mmHg)**		
**Ankle Systolic Pressure (mmHg)**		
**Brachial Systolic Pressure (mmHg)**		
**ABPI value**		
**TBPI value**		

Note. ABPI= ankle-brachial pressure index; TBPI= toe-brachial pressure index. ABPI≤0.9 or TBPI≤0.6 indicates peripheral arterial disease. ABPI>1.3 or non-compressible arteries (i.e. >240 mm Hg) indicates arterial calcification.

4.4 Biomechanical Assessment

4.4.1 Range of Motion

	Left Foot	Right Foot
**1st MTPJ**		

Note. MTPJ= metatarsophangeal joint. Range of motion <65° indicates limited joint mobility of the 1st MTPJ.

4.4.2 Foot Deformity

	Left Foot	Right Foot
**Hammer/Claw Toes**$_1$		
**HAV**$_2$		
**Bony Prominences**$_3$ (e.g. prominent metatarsal heads)		
**Charcot Neuroarthropathy**$_4$		
**Other**$_5$ (e.g. forefoot pad atrophy, amputation)		

Note. P= present, A= absent. HAV= hallux abducto valgus. HAV graded in accordance with the Manchester Scale. No deformity= 1, mild deformity= 2, moderate deformity= 3, severe deformity= 4. Foot deformity recorded with the presence of ≥1 foot deformities.

4.4.3 Plantar Pressures

Left foot

Trial: 1 ☐ 2 ☐ 3 ☐

Right foot

Trial: 1 ☐ 2 ☐ 3 ☐

4.5 Footwear Assessment

4.5.1 Fit

Length: *(palpation)* Good (thumbs width) ☐1 Too short (<½ thumb) ☐2 Too long (>1½ thumb) ☐3

Width: *(grasp test)* Good ☐1 Too narrow ☐2 Too wide ☐3

Depth: Good ☐1 Too shallow ☐2

4.5.2 General Features

Fixation: None ☐1 Laces ☐2 Velcro ☐3

Straps/buckles ☐4 Zips ☐5

Forefoot Sole Flexion Point:
at level of MTPJs ☐1 proximal to 1st MTPJ ☐2 distal to 1st MTPJ ☐3

Heel Height: 0-2.5cm ☐1 2.6-5.0cm ☐2 >5.0cm ☐3

Materials: (upper) Leather ☐1 Synthetic ☐2 Mesh ☐3

Other4 ______________

4.5.3 Type

Footwear Style:

Walking shoe	☐1	Ugg-boot	☐6	Court shoe	☐11
Boot	☐2	Backless slipper	☐7	Moccasin	☐12
Slipper	☐3	Surgical/bespoke	☐8	Thong/flip-flop	☐13
Sandal	☐4	Oxford shoe	☐9	Mule	☐14
Athletic shoe	☐5	High heel	☐10	Other15 ______________	

4.5.4 Condition

Age of Shoe: 0-6 months ☐1 6-12 months ☐2 >12 months ☐3

4.6 Dermatological Assessment

4.6.1 Skin Pathology

	Left foot	Right foot
**Hyperkeratosis**[1]		
**Heloma Dura**[2]		
**Uraemic Pruritus**[3]		
**Xerosis**[4]		
**Calciphylaxis**[5]		
**Other**[6]		

Note. P= present, A= absent. Skin pathology recorded with the presence of ≥1 skin pathologies. Xerosis graded in accordance with the Xerosis Severity Scale. Mild= 1-2, moderate= 3-4, severe= 5-6.

4.6.2 Nail Pathology

	Left foot	Right foot
**Half-and-Half Nail**[1]		
**Absent Lunula**[2]		
**Onychomycosis**[3]		
**Onychocryptosis**[4]		
**Onychauxis**[5]		
**Other**[6]		

Note. P= present, A= absent. Nail pathology recorded with the presence of ≥1 nail pathologies.

4.7 Foot Health Care Behaviours/Podiatry Attendance

4.7.1 Provision of Foot Care

Daily Foot Care

Inspection of Feet: Yes ☐1 No ☐2

Other

Avoid Barefoot Walking: Yes ☐1 No ☐2

Flexibility to Reach Feet: Yes ☐1 No ☐2

Treatment of nails and skin lesions: Yes ☐1 No ☐2

4.7.2 Podiatry Attendance

Seen a Podiatrist: Yes ☐1 No ☐2

Number of times attended a podiatrist in the last 12 months ____________
