# Supplementary material for: Risk factors for foot ulceration in adults with end-stage renal disease on dialysis: study protocol for a prospective observational cohort study
Source: J Foot Ankle Res. 2015 Sep 18;8:53. doi: 10.1186/s13047-015-0110-9 (PMC4575467; doi:10.1186/s13047-015-0110-9)
Supplement: Additional file 2: — Data collection form: Prospective data (12-month follow-up). Data collection form is designed to obtain primary and secondary outcome data at the 12-month follow-up. (PDF 145 kb) [file 13047_2015_110_MOESM2_ESM.pdf]

Data Collection Form: Prospective Data

(12-month follow-up)

Baseline assessment date: ____________Follow-up assessment date:____________

1. Recurrent Foot Ulceration

1.1 Baseline foot ulcer?

**Left foot**	Yes	☐1	No	☐2	Location(s):____________
**Right foot**	Yes	☐1	No	☐2	Location(s):____________

If no, continue to Section 2 (New Foot Ulceration)...

1.2 Healing of baseline foot ulcer?

**Left foot**	Yes	☐1	No	☐2	Location(s):____________
**Right foot**	Yes	☐1	No	☐2	Location(s):____________

Date of healed foot ulcer(s): ____________

1.3 Recurrent breakdown of baseline foot ulcer?

**Left foot**	Yes	☐1	No	☐2	Location(s):____________
**Right foot**	Yes	☐1	No	☐2	Location(s):____________

Date of reoccurring foot ulcer breakdown(s): ____________

2. New Foot Ulceration

2.1 New foot ulcer?

**Left foot**	Yes	☐1	No	☐2	Location(s):____________
**Right foot**	Yes	☐1	No	☐2	Location (s):____________

Date of discovered new foot ulcer(s): ____________

3. New Lower Extremity Amputation

3.1 Baseline Lower Extremity Amputation?

**Left foot**	Yes	☐1	No	☐2	Location(s):____________
**Right foot**	Yes	☐1	No	☐2	Location(s):____________

3.2 New Lower Extremity Amputation(s)?

**Left**	Yes	☐1	No	☐2	Location(s):____________
**Right**	Yes	☐1	No	☐2	Location (s):____________

Date of new lower extremity amputation(s):____________

If no, continue to Section 4 (Episodes of Infection of the Foot or Lower Extremity)…

3.3 Reason for Lower Extremity Amputation?

Trauma	☐1	*Do not record*
Tumour	☐2	*Do not record*
Infected foot ulcer	☐3	
Peripheral arterial disease/gangrene	☐4	
Osteomyelitis	☐5	
Other____________	☐6	

4. Episodes of Infection of the Foot or Lower Extremity

4.1 Lower extremity infections?

**Left**	Yes	☐1	No	☐2	Type:____________
**Right**	Yes	☐1	No	☐2	Type:____________

5. Episodes of Osteomyelitis

5.1 Osteomyelitis episode(s)?

**Left foot**	Yes	☐1	No	☐2
**Right foot**	Yes	☐1	No	☐2

6. Foot-Related Hospitalisations

6.1 Foot-Related Hospital Admission(s)?

Yes ☐1 No ☐2

Date of hospital admission(s):____________

Date of hospital discharge(s): ____________

If no, continue to Section 7 (Revascularisation Procedure of the Lower Extremity)…

6.2 Reason for Hospital Admission?

Infected foot ulcer	☐1
Lower extremity amputation	☐2

Lower extremity revascularisation procedure ☐3
Peripheral arterial disease/gangrene ☐4
Cellulitis ☐5
Osteomyelitis ☐6
Other____________ ☐7

6.3 Foot-Related Treatments/Procedures Received During Hospital Admission?

Prescription of antibiotics ☐1
Wound care/management ☐2
Lower extremity revascularisation procedure ☐3
Surgical debridement ☐4
Issued an offloading device (e.g. TCC, CAM) ☐5
Podiatry treatment ☐6
Lower extremity amputation ☐7
Other____________ ☐8

7. Revascularisation Procedure of the Lower Extremity

7.1 Lower Extremity Revascularisation Procedure?

Yes ☐1 No ☐2 Type:____________

8. New Podiatry Interventions

8.1 New Attendance to Podiatry (since baseline)?

Yes ☐1 No ☐2 N/A ☐3

8.2 New Podiatry Interventions Received (since baseline)?

Yes ☐1 No ☐2

If no, continue to Section 9 (Kidney Transplantation)…

8.3 Types of New Podiatry Interventions?

Nail/callus reduction ☐1
Wound care/management ☐2
Prescription of foot orthoses ☐3
Issued an offloading device (e.g. TCC, CAM) ☐4
Padding/strapping ☐5
Footwear education ☐6
Foot care education ☐7
Other____________ ☐8

9. Kidney Transplantation

9.1 Kidney Transplant?

Yes ☐1 No ☐2

Date of kidney transplant:____________

10. Mortality

10.1 Death during study period?

Yes ☐1 No ☐2

Date of death:____________

10.2 Primary cause(s) of death:________________________

10.3 Secondary cause(s) of death:________________________

10.4 Foot-related death?

Yes ☐1 No ☐2
